# Reducing childhood mortality extends mothers’ lives

**DOI:** 10.1038/s41598-024-61217-w

**Published:** 2024-05-09

**Authors:** Matthew N. Zipple

**Affiliations:** Laboratory for Social Animal Evolution and Recognition, Department of Neurobiology and Behavior, Cornell University, Ithaca, NY USA

**Keywords:** Human behaviour, Risk factors, Public health

## Abstract

During the twentieth century, childhood mortality was dramatically reduced globally, falling by more than 90% in the United States and much of Europe. Total fertility also fell, with the combined result that many parents who otherwise would have experienced the loss of a child were spared the trauma and negative health consequences that accompany such a loss. Here I use mathematical modeling to argue that the reduction in the frequency of child death that occurred in the twentieth century indirectly led to a substantial reduction in female mortality, resulting in an extension of female lifespan. I estimate that the reduction in maternal bereavement in the US during the twentieth century indirectly increased mean female lifespan after age 15 by approximately 1 year. I discuss implications for our understanding of the persistence of the sex gap in longevity and approaches to improving maternal health outcomes in countries that still face high levels of childhood mortality.

## Introduction

**Figure 1:**
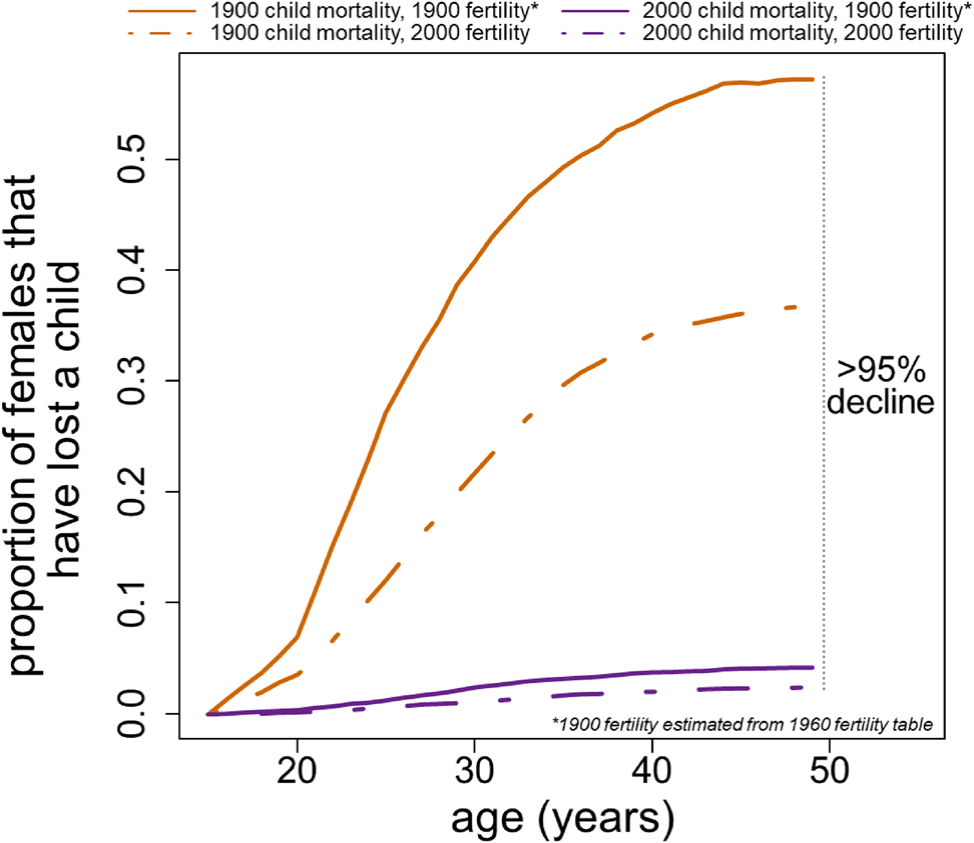
The proportion of females that experience child loss is dramatically reduced when childhood mortality is reduced from 1900-era levels to 2000-era levels. An additional decrease is achieved when total fertility is also reduced to 2000-era levels.

Over the course of the twentieth century, human life expectancy increased dramatically with the global average more than doubling^1^. Much of the total extension in lifespan can be attributed to a decline in childhood mortality. For example, in the United States in 1900, > 18% of children died before their 5th birthday, and 21% died before age 15^2^. In 2000, those same numbers stood at 0.8% and 1%, respectively^2^. Over the same period total fertility rates fell substantially; in the US, total fertility declined from 3.85 children in 1900 to 2.05 children in 2000^3^. The combination of the astounding reductions in child mortality and total fertility resulted in an estimated 96% reduction in the proportion of females that lost a child during their reproductive lives (Fig. 1, below).

This reduction in the frequency of child loss has likely had substantial health and survival implications for parents, especially mothers. The loss of a child before adulthood has been shown across several societies (with varying access to modern medicine) to have adverse impacts on the health of mothers, including striking increases in maternal mortality risk^4–13^. For example, a study spanning nearly 200 years (1800–1996) of demographic, technological, and medical transitions in Iceland found a consistent effect of child death on future maternal death, with mothers who lost a child being 1.3–1.6 times as likely to die as compared to their siblings who had not lost children^13^. This bereavement-induced increase in mortality appears to afflict mothers of all reproductive ages (~ 15–49) and can extend for decades after the death of the child occurs^13^.

Several pieces of evidence point towards the loss of a child having a causal impact on future maternal death. First, if both the child and maternal deaths could be explained by shared environmental risks, we would expect fathers to bear this risk as well. Yet, across studies there is a consistently weaker or non-existent relationship between child death and future death of fathers^5,9,13^. The Icelandic study was able to partially control for shared genetic and environmental risk between child and mother by employing a matched-sibling design^13^. What’s more, mothers in Denmark who lost children were at especially great risk of death by unnatural causes^5^ and experienced an increased risk of myocardial infarction^14^, both results that are consistent with an outsized role of maternal psychological and physical stress in the deaths of bereaved mothers. Perhaps most strikingly, a study from Sweden shows that mothers are at an especially high risk of death on the anniversary of the death of their child^10^.

Given the apparent causal relationship between child loss and premature maternal death, it follows logically that the massive reduction in the proportion of US mothers experiencing child loss that occurred during the twentieth century should have had substantial positive downstream effects on maternal survival and lifespan. The purpose of this paper is to identify the follow-on effects of reductions in childhood mortality and total fertility in the United States from 1900 to 2000 on (a) the frequency of maternal bereavement, (b) female mortality, and (c) female life expectancy.

## Mathematical methods and results

In 1900, two groups of reproductively aged (15–49 year-old) females of similar size existed in the US—those that had lost a child aged 0–17 (“bereaved females”) and those who had not (“non-bereaved females”). As a result of the astounding reduction in the proportion of females experiencing the loss of a child during the twentieth century, nearly all females were shifted into a single group that never experienced child loss. Thus, even in the absence of any other technological or social changes, the twentieth century reduction in experienced maternal bereavement would have caused the average mortality risk for all females in the United States to decline to almost perfectly match the mortality risk of non-bereaved 1900-era females.

The average observed mortality at age *x* (*q*_*x*_) for a population of reproductively-aged females is the weighted average of mortality faced by bereaved and non-bereaved females:

1 $${q}_{x}=\left(1-{p}_{x}\right)\cdot {q}_{x}\cdot \gamma +{p}_{x}\cdot {q}_{x}\cdot \gamma \cdot {\Delta }_{br}$$

where *p*_*x*_ is the proportion of females of age *x* that are bereaved, $\gamma$ is a value [0, 1] that modifies the observed total observed mortality—such that ${q}_{x}\gamma$ is equal to the experienced mortality of non-bereaved females—and ${\Delta }_{br}$ is the relative risk or hazard ratio (HR) faced by bereaved females relative to non-bereaved females.

Solving Eq. (1) for the inferred mortality experienced by non-bereaved females (${q}_{x}\gamma )$ yields

2 $${q}_{x}\gamma =\frac{{q}_{x}}{1+{p}_{x}\cdot {\Delta }_{br}-{p}_{x}}$$

And the mortality experienced by bereaved females is ${\Delta }_{br}{\cdot q}_{x}\gamma$.

Thus, three parameters together allow us to infer the experienced mortality of bereaved and non-bereaved females at any given time: (1) the observed age-specific mortality for all females in a population (*q*_*x*_), (2) the relative mortality risk experienced by bereaved females as compared to non-bereaved females (${\Delta }_{br}$), and (3) the proportion of females of a given age that are expected to be bereaved (*p*_*x*_).

1. To estimate age-specific mortality in 1900, I applied a geometric smoothing function to mortality estimates from period tables for 1900–1902 (hereafter “1900”) published by the CDC (^2^, see Fig. S1 this paper).
2. I estimated ${\Delta }_{br}$ based on the existing literature. To my knowledge there are 5 studies that have estimated the hazard ratio of child loss on future maternal survival^5,8,9,11–13^. From this set I extracted two estimates of the hazard ratio of child loss that optimize along different axes of potential relevance. First, a study of Icelandic females living from 1800 to 1996 (HR = 1.36), is likely to best approximate the healthcare environment available to bereaved females in the US in 1900^13^. This is also the most complete and best-controlled study of the effects of child loss on maternal mortality. Second, to leverage the collective power of these studies, I took the average of their 5 reported hazard ratios (geometric mean = 1.48, see Supplement) and considered this as an alternative estimate for ${\Delta }_{br}$ (Table 1).
3. To estimate the proportion of US females in 1900 and 2000 that experienced bereavement at any given age, I built a computer simulation of female lives using period tables of female mortality and fertility using publicly available datasets^15–17^. Age-specific fertility data were not available for 1900, so I instead used fertility data from 1960 as an estimate^15^. These provide a reasonable proxy for age-specific fertility in 1900, as total fertility was comparable in the US in 1960 (~ 3.6 children per female)^15^ and 1900 (~ 3.9)^3^. To the extent that use of the 1960 data biases the below results, it should be in the conservative direction as it would result in a slight underestimation of the proportion of females experiencing the death of a child in 1900. I simulated 10,000 female lives from ages 15–49 and tracked the proportion of surviving females that had ever experienced child loss.

**Table 1:** Studies that estimate the mortality hazard ratio faced by mothers who have lost a child.

Country	Years included	Hazard ratio estimate	References
United States	1979–1990	2.33	Espinosa and Evans, 2013^11^
Iceland	1800–1996	1.36	Valdimarsdóttir et al., 2019^13^
Sweden	1980–2002	1.31^a^	Rostila et al., 2012a^9^
Israel	1964–2010	1.18	Schorr et al., 2016^12^
Denmark	1980–1997	1.43	Li et al., 2003^5^
		Geometric mean = 1.48	

^a^Reports annualized relative risk from Cox proportional hazards models that control for parental age.

The proportion of simulated 1900-era females that had experienced child loss increased rapidly from 0 at age 15 to 0.27 at age 25 and reached a maximum of 0.57 at age 49 (Fig. 1, solid orange curve). Thus, a majority of females that survived to be 49 years old in the US in 1900 would have experienced the death of a child, and many would have experienced multiple such deaths.

I then substituted child mortality rates and age-specific fertility rates from 2000 into the same simulation (Fig. 1, dashed purple curve). The result was a dramatic reduction (> 95%) in the proportion of females experiencing bereavement, with a new maximum value of 0.025 at age 49. These simulated estimates are consistent with empirical data. Today, 1.7–3.8% of 45–49-year-old women living in the US and Western Europe have experienced the loss of a child^16^. The estimate for the cumulative proportion of females experiencing child loss in the US in the 1900s is consistent with measures from least developed countries today: more than half of 45–49 females report experiencing the loss of at least one child in 21 least developed countries^16^.

Together, these estimates allow for the inference of age-specific mortality affecting females in the US in 1900 that were not experiencing bereavement (see Eq. 2). Complete elimination of childhood mortality would effectively shift *all* females into this non-bereaved category, with the result being a corresponding reduction in population-level female mortality rates and an increase in female life expectancy.

**Figure 2:**
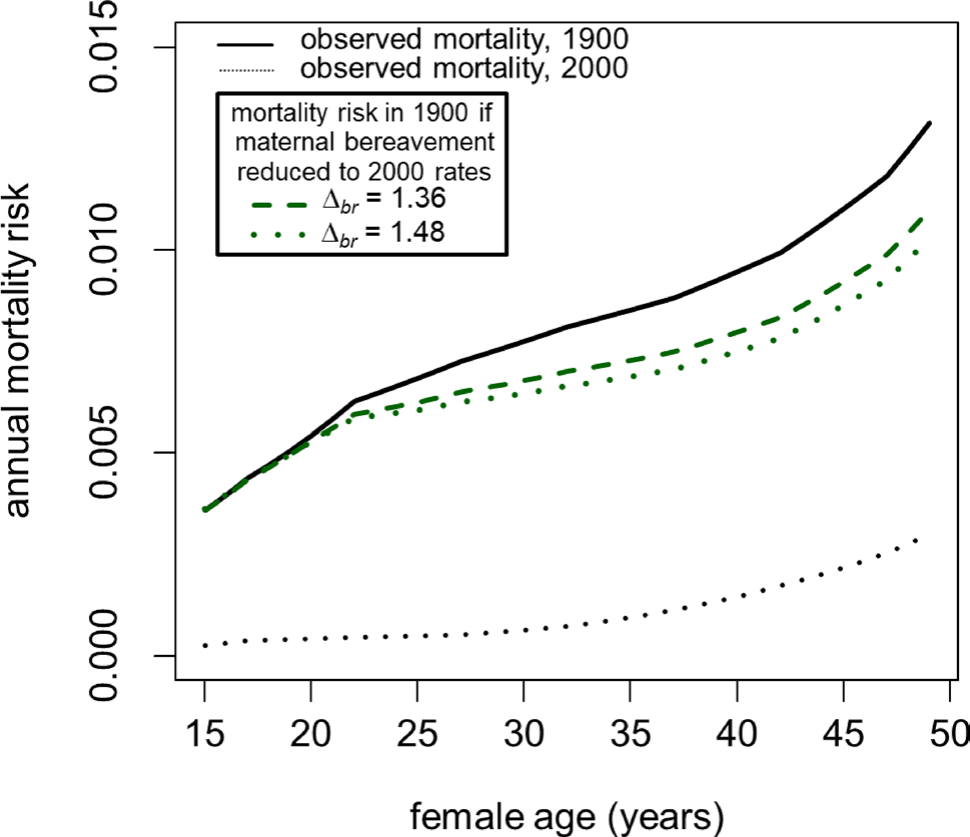
The inferred rates of mortality for non-bereaved females in 1900 are substantially lower than the observed mortality rates. Elimination of child loss would have shifted all females into the non-bereaved category, resulting in an overall reduction in population mortality.

Childhood mortality has not been eliminated in the US. So, to estimate the change in mortality that we can ascribe to the reduction in child loss, I evaluated Eq. (1), replacing *p*_*x*_ with my age-specific estimates of the proportion of females experiencing bereavement in 2000 (Fig. 1, purple dashed line). I then calculated expected population-level mortality (Fig. 2, dashed lines) and lifespan metrics for the two values of ${\Delta }_{br}$.

For both modeled scenarios of ${\Delta }_{br}$, the reduction in child loss that occurred in the US over the twentieth century yielded a substantial reduction in female mortality. Under the more conservative scenario (${\Delta }_{br}=1.36$) a 40-year-old female living in an environment that is identical in every way to 1900, except that her risk of experiencing the death of a child has been reduced to 2000-era levels, enjoys a 16% reduction in overall mortality risk as a result. Under the more aggressive scenario (${\Delta }_{br}=1.48$) this reduction in mortality risk is estimated to be 20%. As a result of these reductions in mortality, total experienced mortality for 15–49 year-olds declines by 11% to 15%. From 1900 to 2000, total mortality for 15–49 year-olds was reduced by 84% in the US as a result of the combination of all life-extending advances developed during that period. Thus, the near-elimination of the experience of child loss may explain a substantial portion of the total reduction in mortality faced by 15–49 year-old US females in the twentieth century.

Finally, I used these mortality estimates to assess the magnitude of the increase in female lifespan after age 15 that can be attributed to the reduction in frequency of child loss. Converting mortality risk to life expectancy metrics indicates that that the twentieth century reduction in the frequency of child loss in the US may have increased mean female lifespan after age 15 by 0.9 years (${\Delta }_{br}=1.36$) to 1.2 years (${\Delta }_{br}=1.48$). The estimated effect of reducing the frequency of child loss on median female lifespan after age 15 is somewhat smaller, ranging from 0.8 to 1.0 years.

### Research ethics approval

This manuscript does not make use of any individual-level or protected data. This study did not involve human participants or animals and research ethics approval was therefore not required.

## Discussion

I have shown here that female lifespan is substantially extended when child mortality and total fertility are dramatically reduced. I have estimated that the reduction in frequency of child loss experienced by mothers in the twentieth century in the United States led to a previously undocumented increase in female lifespan after age 15 of as much as 0.9–1.2 years. Notably, while reductions in total fertility play a role in reducing the proportion of females that experience child loss, the vast majority of the reduction in child loss achieved across the twentieth century can be attributed to reductions in childhood mortality (Fig. 1, compare two purple curves).

These results give insight into the persistence of the sex gap in life expectancy. Even as life expectancy has increased in the US, females have continued to live longer than males, with only a minor narrowing of the gap during the twentieth century^17^. While fathers may face some increased risk of mortality due to bereavement, studies have repeatedly demonstrated that any such effect is small, and much smaller than the effect of child loss on maternal survival^5,9,13^. Thus, we would not expect the tremendous decline in the frequency of child loss to have substantially increased male lifespan alongside female lifespan. Even if other factors worked to narrow the longevity sex gap across the twentieth century, this sex-specific increase in lifespan due to the reduction in the frequency of maternal bereavement may have effectively increased the sex gap by a about a year over the same period.

Maternal bereavement is not the only form of bereavement that leads to increased mortality. The death of a spouse^18–22^ or parent^23^ have been linked to increased mortality in the years following death. As mortality has declined for all age groups in the past century, it follows that experiencing these other types of bereavement should become less frequent (or at least shifted later in life), leading to a reduction in the overall impact of bereavement on mortality and lifespan. What’s more, the impacts of these distinct effects should interact multiplicatively—if mothers live longer because they experience less bereavement, their spouses, siblings, and surviving children are less likely to experience bereavement as well or to experience that bereavement later in life. The current analysis suggests that similar examinations of these other forms of bereavement and their interactions would likely explain additional fractions of the reduction in mortality that has characterized the last century.

### Implications for practice and/or policy

Parents in less- and least-developed nations still face relatively high levels of under-5 mortality and high levels of parental bereavement as compared to the United States. From 2015 to 2020, under-5 mortality in least-developed nations stood at 6.8%^24^. In the extreme case, mothers in 20 African countries with relatively high child mortality and annual fertility report cumulative levels of bereavement comparable to my estimate for women in the US in 1900^16^. The results of the present analysis further underscore the reverberating benefits that reducing childhood mortality produce in a society. While the intended beneficiaries of efforts to reduce child mortality are generally the children themselves (see, e.g. Millennium Development Goal 4^25^), it is clear that reducing childhood mortality also has downstream positive impacts on maternal health and lifespan.

### Limitations of the model

As in any modeling paper, I needed to make several central assumptions that influenced the results of this investigation. Most importantly, I assume that hazard ratios following the loss of a child are (1) constant across the reproductive lifespan and (2) constant across time and space. Each of these assumptions has some empirical support in published work, but may not reflect the complexity and cultural-specificity of these phenomena. For example, it is possible that the negative consequences of bereavement for mothers were less severe when expectations of child loss were higher, a possibility that would reduce the positive effect of reductions in child mortality. However, the best data available to assess this possibility instead point to a near-constant hazard ratio over two centuries of development in Iceland^13^. To generate a reasonable range in variation in the magnitude of the hazard ratio estimate I consider two possible hazard ratios, but it is possible that the true effect of maternal bereavement in the context considered here or in other contexts may fall outside of this range.

## Conclusions

The dramatic reduction in the frequency with which mothers experience bereavement due to child loss that has occurred in the last century is likely to have indirectly extended female lifespan. In the United States, I estimate that this reduction in the frequency of child loss has indirectly extended maternal lifespan by about 1 year. This result has direct implications for policy makers. When policies prioritize reducing the experience of child loss—both through reductions in child mortality and by increasing access to family planning resources that may lead to reductions in total fertility—they generate a hidden but important downstream impact on the health and survival of mothers that are spared the trauma of losing a child. This study is parameterized from data that have largely come from Europe and North America. Additional studies of the impact of maternal bereavement on maternal mortality from a wider range of cultures and levels of development are critical for understanding either consistency or variation in the magnitude of this effect.

### Supplementary Information

Supplementary Information.

## Data Availability

Data and code underlying these analyses and results are available from the Cornell University eCommons Repository. https://doi.org/10.7298/1r0d-tc78.
