# Supplementary material for: Reducing childhood mortality extends mothers’ lives
Source: Sci Rep. 2024 May 9;14:10649. doi: 10.1038/s41598-024-61217-w (PMC11082133; doi:10.1038/s41598-024-61217-w)
Supplement: Supplementary file 1 — Supplementary Information. [file 41598_2024_61217_MOESM1_ESM.docx]

**Supplemental Material:**


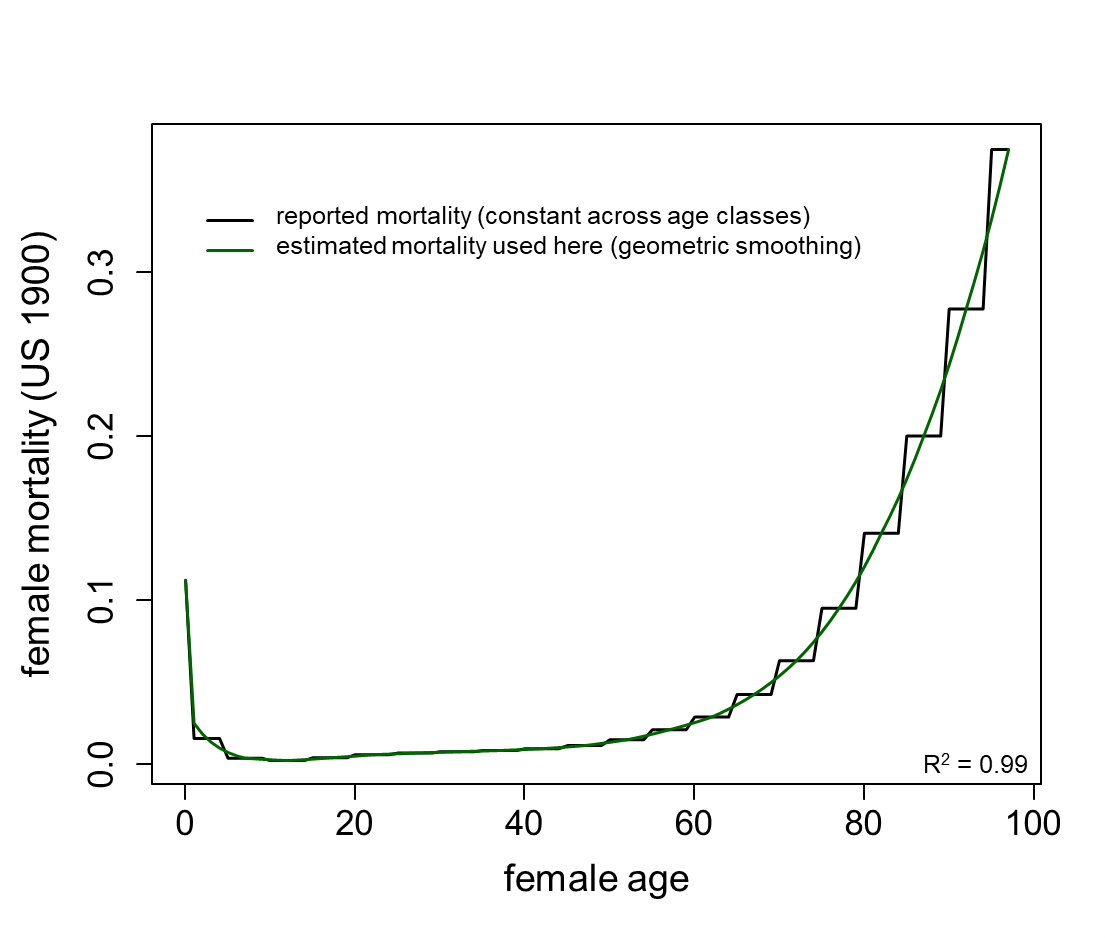


**Figure S1.** The geometric smoothing function I applied to age-specific female mortality for these analyses. Data reported by the CDC (black) assumes a constant mortality rate within a 5-year age class, an assumption that is unlikely to be true. To more realistically model maturation and senescence in my models, I’ve assumed that mortality declines and rises smoothly (dark green).
